# Modeling the effects of genetic- and diet-induced obesity on melanoma progression in zebrafish

**DOI:** 10.1242/dmm.049671

**Published:** 2023-01-20

**Authors:** Emily Montal, Dianne Lumaquin, Yilun Ma, Shruthy Suresh, Richard M. White

**Affiliations:** ^1^ Cancer Biology and Genetics Program, Memorial Sloan Kettering Cancer Center, New York, NY 10065, USA; ^2^ Weill Cornell/Rockefeller/Sloan Kettering Tri-Institutional MD-PhD Program, New York, NY 10065, USA

**Keywords:** Obesity, Melanoma, Zebrafish, Agrp, Diet, Genetics

## Abstract

Obesity is a rising concern and associated with an increase in numerous cancers, often in a sex-specific manner. Preclinical models are needed to deconvolute the intersection between obesity, sex and melanoma. Here, we generated a zebrafish system that can be used as a platform for studying these factors. We studied how germline overexpression of Agrp along with a high-fat diet affects melanomas dependent on BRAF^V600E^ and loss of p53. This revealed an increase in tumor incidence and area in male, but not female, obese fish, consistent with the clinical literature. We then determined whether this was further affected by additional somatic mutations in the clinically relevant genes *rb1* or *ptena/b*. We found that the male obesogenic effect on melanoma was present with tumors generated with BRAF;p53;Rb1 but not BRAF;p53;Pten. These data indicate that both germline (Agrp) and somatic (BRAF, Rb1) mutations contribute to obesity-related effects in melanoma. Given the rapid genetic tools available in the zebrafish, this provides a high-throughput system to dissect the interactions of genetics, diet, sex and host factors in obesity-related cancers.

## Teaser

**Editor's choice:** Obesity incidence is rising, resulting in increased occurrence of obesity-related cancers often occurring in a sex-specific manner. Here, we developed a zebrafish model to investigate the intersection between obesity, sex and melanoma.

## INTRODUCTION

There has been a sharp rise in the number of overweight and obese people in the USA and worldwide. The Centers for Disease Control and Prevention reported in the National Health and Nutrition Examination Survey 2017-March 2020 pre-pandemic data file that 41.9% of Americans over the age of 20 were obese or severely obese, an increase from 30% in 2000 (National Health Statistics Reports, 2021). According to the World Health Organization (WHO), more than 39% of adults are overweight or obese globally, and it is estimated that 23% of adults were obese in the WHO European region in 2016 (World Health Organization, 2022). As the incidence of obesity increases, so does the prevalence of diseases that are associated with obesity. These include heart disease, type 2 diabetes, metabolic syndrome and several cancers, such as breast, colorectal and pancreatic cancer, and melanoma (Renehan et al., 2008; Wolk et al., 2001).

Melanoma is the most lethal form of skin cancer. It is derived from melanocytes or their neural crest precursors in the skin. As melanoma invades deeper into the skin, the cells encounter several microenvironmental cell types including adipocytes. These adipocytes contribute to melanoma invasiveness by providing fatty acids as fuel for growth (Zhang et al., 2018). Obesity is well known to increase adipocyte numbers and sizes, and thus it is likely that the crosstalk between melanoma and adipocytes would be enhanced in obese individuals (Björntorp and Sjöström, 1971; Verboven et al., 2018). Clinically, the effect of obesity on melanoma is suggested, but the data are not entirely clear. For example, clinical studies suggest that there is an increased hazard ratio for melanoma associated with obesity, but this is only in males (Karimi et al., 2016; Sergentanis et al., 2013). Further adding to this complexity, targeted therapy has been shown to be more effective in obese males than in their lean counterparts. This does not occur in females, suggesting a sex-dependent effect of obesity on melanoma (McQuade et al., 2018). Preclinical animal studies using both genetic and diet models of obesity in mice demonstrate that there is an increase in xenograft tumor size in the obese setting (Brandon et al., 2009; Pandey et al., 2012; Ringel et al., 2020). These studies, however, do not address the effect of sex in these animals, nor do they test the effect of obesity on response to therapy. Furthermore, studies focusing on the systemic versus local effect of obesity on melanoma have been limited. Therefore, there is a critical need to develop preclinical models to further clarify the questions surrounding obesity, sex and melanoma.

Because the effects of obesity on cancer occur within the context of host physiology, it is important to study this in model organisms. The mouse is the most traditional model for this work, given its amenability to genetic manipulation, robust cancer models and ease of dietary interventions. However, there are limitations to mice in terms of the number of genetic manipulations that can be done, often requiring large numbers of crosses to gain germline alleles, and challenges in performing *in vivo* unbiased screens. In addition, detailed *in vivo* imaging remains difficult in the mouse outside of specialized equipment.

The zebrafish has emerged as an important model organism in cancer biology. The major advantage of the model is that it is highly amenable to large-scale and rapid genetic manipulation (using CRISPR or cDNA screens), allows for detailed *in vivo* imaging (especially in the *casper* strain), can be used for small-molecule screens and has a wide variety of cancer models available (Heilmann et al., 2015; Patton et al., 2021; Zhang et al., 2012). For melanoma, it has been widely used to study the effects of BRAF or NRAS (key initiating events in melanoma) and has uncovered important developmental and microenvironmental influences on these tumors (Kaufman et al., 2016; Zhang et al., 2018).

Although less studied, the zebrafish has also increasingly been used to study host physiology and disease in the context of cancer. This includes metabolite tracing and inter-organ crosstalk (Naser et al., 2021). Several models of obesity have been generated in the zebrafish, using either diet or genetic manipulation, and these closely resemble key aspects of the condition in humans (Landgraf et al., 2017; Song and Cone, 2007; Zang et al., 2018). For example, Agouti-related protein (AGRP) is a neuropeptide secreted in the hypothalamus as part of the central melanocortin signaling system that regulates food intake (Ollmann et al., 1997). Clinically, the central melanocortin system is the pathway with the most mutations observed in genetically obese patients (Loos and Yeo, 2022; Mendes de Oliveira et al., 2021). Although obese patients with mutations in AGRP have not been identified, there is increased expression of AGRP in response to obesity in both mice and humans (Katsuki et al., 2001; Shutter et al., 1997). Ubiquitous overexpression of AGRP has been shown to increase appetite, resulting in increased weight and obesity in mice (Graham et al., 1997). The central melanocortin system and AGRP are highly conserved between mammals and zebrafish, and overexpression of the peptide leads to a similar phenotype in this organism (Song and Cone, 2007; Song et al., 2003). The cells affected in the zebrafish obesity models continue to be characterized. These affected cell types at a minimum pertain to white adipocytes (i.e. subcutaneous or visceral adipocytes), because the fish are not thought to have thermogenic brown adipocytes. Other organs affected by obesity, including the liver and skeletal muscle, are also present in this organism, implying that it can be readily studied in the context of cancer. In this study, we utilize these unique attributes of the zebrafish to investigate the intersection of germline and somatic genetics in a melanoma model of obesity. The methods described here can be leveraged in future studies for larger-scale discovery-based efforts to uncover new mediators of this crosstalk with relevance to the human diseases.

## RESULTS

### Agrp overexpression promotes obesity in *casper* zebrafish

**Fig. 1.**
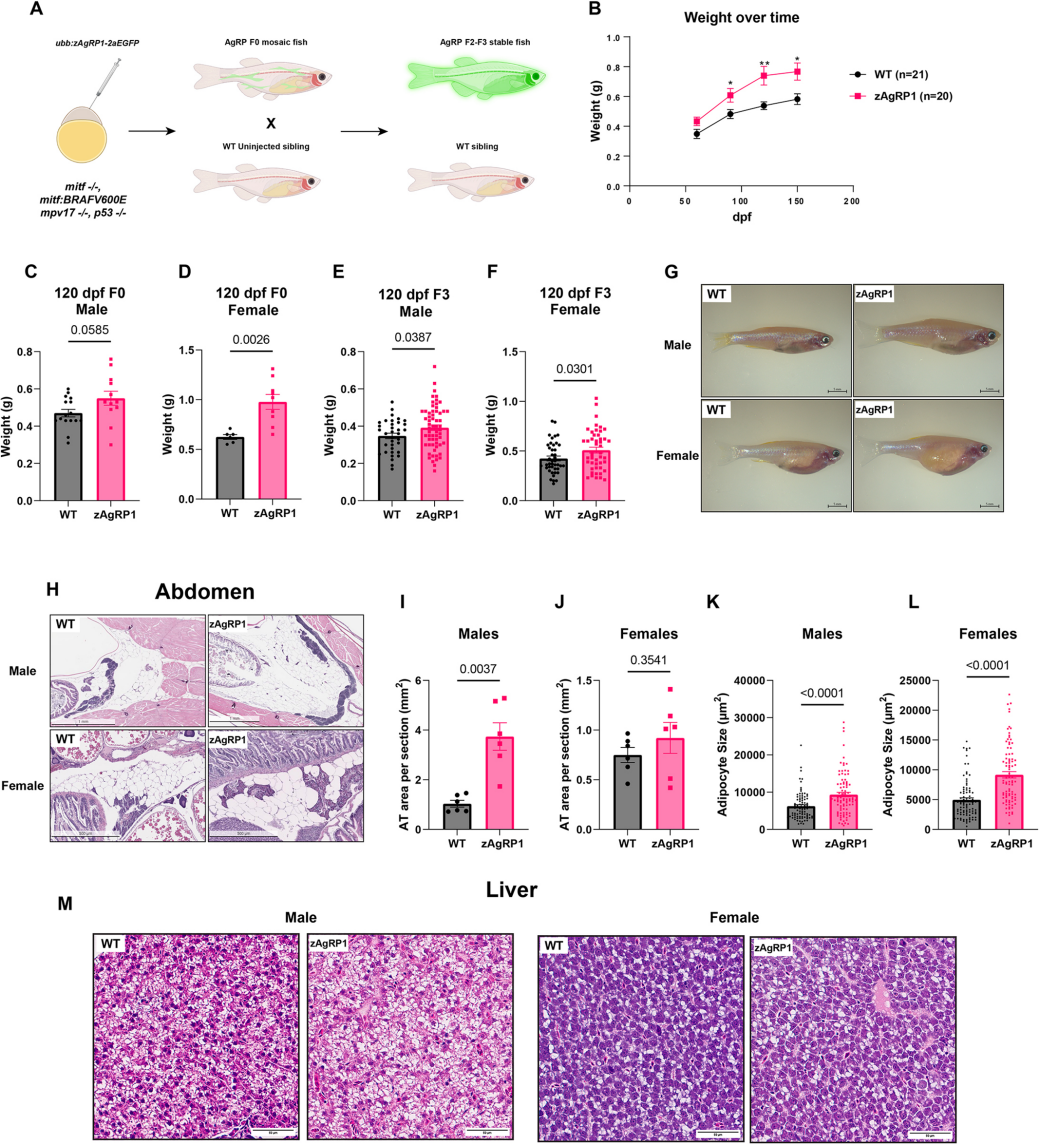
**Agrp overexpression promotes obesity in *casper* zebrafish.** (A) Schematic of generation of zebrafish *agrp* cDNA (zAgRP1)-overexpressing *casper* zebrafish (*mitfa:*BRAF^V600E^, *p53*^−/−^, *mitfa*^−/−^, *mpv17*^−/−^). Created with Biorender.com. WT, wild type. (B) Weight of both male and female F0 fish combined over 6 months. (C,D) Weights of male (C) and female (D) F0 mosaic fish at 120 days post-fertilization (dpf). Fish were separated into equal numbers at 1 month, and their weights were measured at the indicated time points. *n*=5-10 fish per genotype per biological replicate. Data are the averages of three biological replicates. (E,F) Weights of male (E) and female (F) fish of the F3 stable line at 120 dpf. Data are the weights of all the fish from two clutches from separate single male and female pairs of fish across six separate tanks. *n*=35-60 fish per group. zAgRP1 fish and wild-type siblings were housed in the same tanks and identified via GFP fluorescence. (G) Representative images of male and female zAgRP1 fish and wild-type siblings. (H) Histology of abdomen from 7-month-old F3 male and female zAgRP1 or wild-type siblings. Representative images are shown. Fish were fixed in PFA, sectioned and stained with H&E. (I,J) Quantification of adipose tissue (AT) per section from male (I) and female (J) fish. Three fish per sex per genotype and two sections per fish were analyzed. (K,L) Quantification of individual adipocyte size of male (K) and female (L) fish. (M) Histology of liver from 7-month-old male F3 male and female fish. Fish were fixed in PFA, sectioned and stained with H&E. Three fish per genotype per sex were sectioned for histology. *P≤0.05, **P≤0.01. Welch's *t*-test. Scale bars: 5 mm (G), 1 mm (H, top row), 500 μm (H, bottom row), 50 μm (M).

Previous studies have shown that overexpression of the orexigenic peptide AgRP1 (Agrp) in zebrafish results in fish that are overweight with hypertriglyceridemia and fatty liver (Song and Cone, 2007). We generated a plasmid with the *ubiquitin B* (*ubb*) promoter, which drives the expression of zebrafish *agrp* cDNA (zAgRP1), followed by a 2aGFP to help visualize its expression (Fig. S1A) (Mosimann et al., 2011; Song et al., 2003). We first confirmed the obesogenic effects of zAgRP1 in fish without melanoma by injecting the plasmid into *casper* (*mpv17^−/−^, mitfa^−/−^*) fish to generate mosaic zAgRP1-overexpressing fish (Fig. 1A). We chose to utilize the *casper* line of zebrafish because the fish are optically transparent, which allows for high-resolution imaging and tracking of melanomas (D'Agati et al., 2017). These fish also contain an inactive *BRAF^V600E^* oncogene in the germline and a p53 (Tp53) mutation, so they will not spontaneously develop melanoma but can be induced to do so rapidly as explained further below. The mosaic fish displayed variable GFP expression (Fig. S1B), which did not appear to substantially differ between sexes. We then outcrossed these fish to generate stable zAgRP1 lines [*Tg(-3.5ubb:zAgRP1-2A-EGFP)*]. Several pairings of a single male zAgRP1 fish and a single female *casper* (*mitfa:*BRAF^V600E^, *p53^−/−^, mitfa^−/−^, mpv17^−/−^*) fish were bred, and clutches from these separate pairings were utilized and averaged for ensuing experiments. We confirmed zAgRP1 overexpression in the F3 generation by quantitative PCR (qPCR) (Fig. S1C). We characterized the obesogenic effects in both mosaic fish and stable lines. We found that in these basal *casper* fish, mosaic overexpression in this line led to an increase in weight in these fish over time, statistically observed at 4 months post-fertilization (mpf) (Fig. 1B). Furthermore, when observing male and female fish separately, we observed that mosaic overexpression of zAgRP1 led to increased weight in females at 4 months and a trend for an increase in weight in males (*P*=0.0585) at this time point (Fig. 1C,D). In the F3 generation, both male and female zAgRP1-overexpressing fish were heavier and appeared larger than wild-type *casper* fish (*mitfa:*BRAF^V600E^, *p53^−/−^, mitfa^−/−^, mpv17^−/−^*) (Fig. 1E-G). We also found that the magnitude of response to zAgRP1 was smaller in the F3 generation than in the F0 generation, particularly in females. This could potentially be due to differences in copy number of zAgRP1 being overexpressed, as mosaic animals tend to have more plasmid copies. We did not observe statistical differences in length, an alternative measure of obesity in zebrafish, in either the mosaic or stable *Tg(-3.5ubb:zAgRP1-2A-EGFP)* fish; however, there was a trend for male zAgRP1 fish being longer than male wild-type fish (Fig. S2A-E) (Song and Cone, 2007). If we calculated the body mass index (BMI) in these fish, female zAgRP1 mosaic and stable fish had a higher BMI than female wild-type mosaic and stable fish, whereas there was no significant difference in BMI between male zAgRP1 and wild-type fish (Fig. S2F-I). This could be due to the fact that male fish seemed to increase in both length and weight, whereas female length did not change, in response to zAgRP1. Taken together, these data demonstrate that zAgRP1 overexpression leads to larger fish in both males and females, with a greater response in females.

To further confirm that overexpression of zAgRP1 in these fish leads to the pathological effects of obesity, we sectioned 5-month-old F3 zAgRP1 fish and sent them for histology. Focusing on adiposity, we found that both male and female zAgRP1 fish had more abdominal adipose tissue (AT) than wild-type control fish (Fig. 1H). When we quantified the total AT per section, we found that there was greater AT area in male zAgRP1 fish than in male wild-type fish (Fig. 1I). We did not see this difference in female fish (Fig. 1J). When we quantified individual adipocyte size, we found that, similar to AT area, there was a significant increase in male fish adipocyte size in zAgRP1 fish compared to wild-type fish (Fig. 1K). Interestingly, we saw an even greater increase in female adipocyte size in zAgRP1 fish compared to wild-type fish (Fig. 1L). Thus, zAgRP1 affected AT in a sex-specific manner, with the effect being both hyperplastic and hypertrophic in males and only hypertrophic in females. These data are intriguing, as the changes in weight observed are more apparent in female fish.

Obesity has clinically been shown to elevate blood triglyceride (TG) levels and blood glucose in patients (Wakabayashi and Daimon, 2012). Therefore, we sought to determine the effects of zAgRP1 on blood metabolites in both male and female fish. We found that, in male fish, there was a significant increase in blood glucose levels but no change in blood TG levels (Fig. S3A,C). In female fish, we found a significant increase in blood TG levels and no change in blood glucose in response to zAgRP1 (Fig. S3B,D). This further demonstrates the sexually dimorphic response to zAgRP1. Finally, we sought to determine the obesogenic effect of zAgRP1 on liver histology. We found that zAgRP1 fish seemed to have fatty liver whereas the wild-type fish did not (Fig. 1M), with a greater effect observed in females. To confirm this, we determined the amount of fat in the liver by examining hepatic Plin2 expression. Plin2 is a protein that surrounds lipid droplets and has previously been used to assess cellular lipid content (Lumaquin et al., 2021). Immunohistochemistry (IHC) of livers taken from zAgRP1 fish demonstrated higher levels of Plin2 protein compared to those in wild-type fish in both males and females (Fig. S3E). Both the Hematoxylin and Eosin (H&E) staining and Plin2 expression results suggest that zAgRP1 alters liver histology, with a more pronounced effect in females. Taken together, these data demonstrate that overexpression of zAgRP1 driven by the *ubb* promoter results in obesity in the *casper* strain of zebrafish, consistent with previous work overexpressing zAgRP1 driven under the actin promoter in zebrafish (Song and Cone, 2007).

### Agrp overexpression increases visceral adiposity

**Fig. 2.**
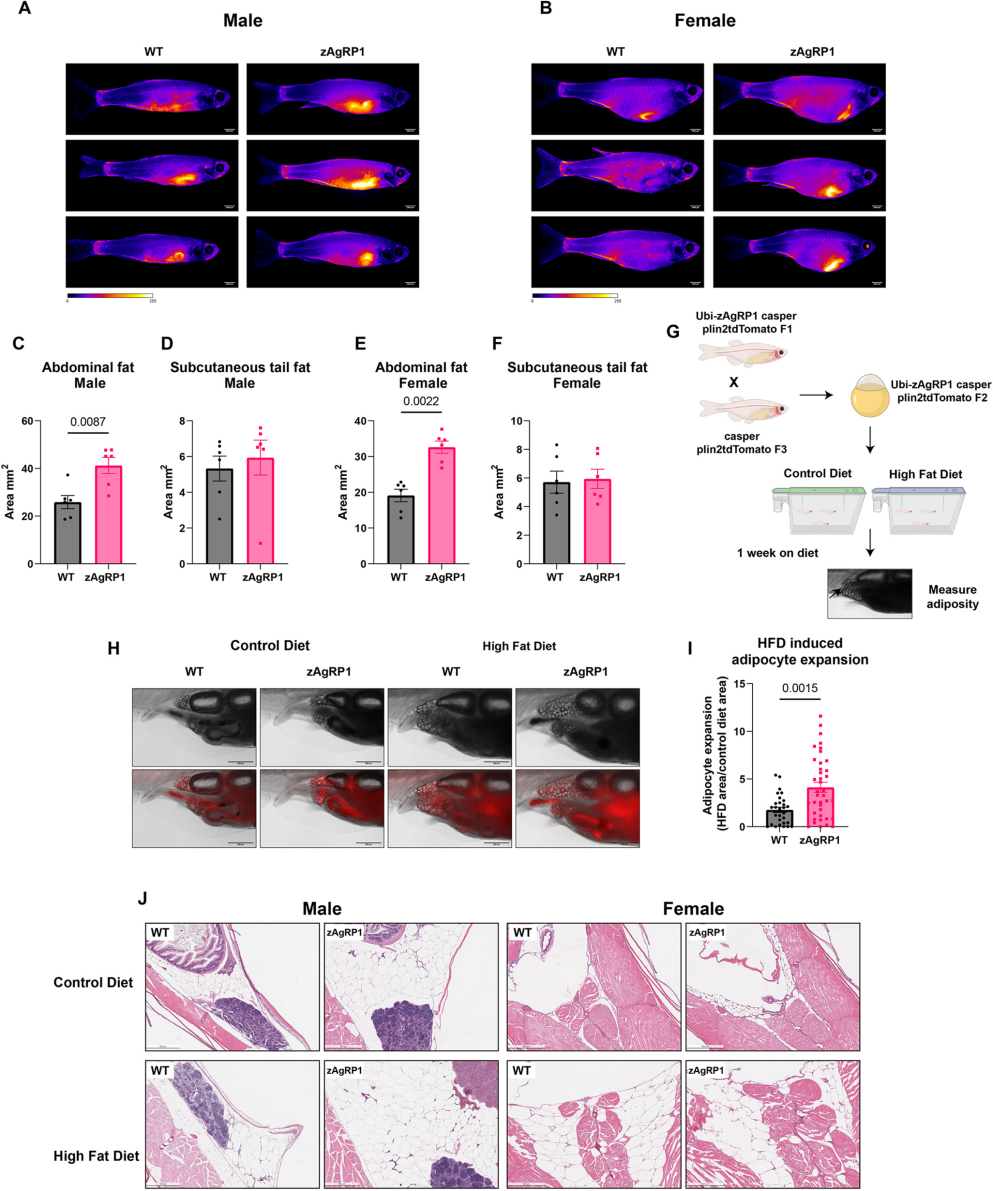
**Agrp overexpression increases visceral adiposity and susceptibility to high-fat diet (HFD).** (A,B) Representative images of male (A) and female (B) BODIPY-stained fish. (C-F) Quantification of visceral abdominal (C,E) and subcutaneous (D,F) fat depots from BODIPY-stained male (C,D) and female (E,F) fish. *n*=6 fish per condition over three biological replicates. (G) Schematic of Plin2tdTomato HFD experiment. 21 dpf zAgRP1 or wild-type Plin2tdTomato fish were put on either a control diet or HFD for 1 week, and visceral adiposity was measured. Created with Biorender.com. (H) Representative images of Plin2tdTomato fish. (I) Quantification of adipocyte expansion. HFD-induced adipocyte expansion was calculated by taking the ratio of the area of adipocyte tissue on the control diet versus HFD for each genotype. *n*≥30 fish per genotype across three biological replicates. (J) Histology of cross-sections of male and female adult quad zAgRP1 fish or wild-type controls on an HFD or control diet for 3 months. Fish were fixed in PFA, sectioned and stained with H&E. Two fish per condition were sent for sectioning. Mann–Whitney test. Scale bars: 2.5 mm (A,B), 500 μm (H,J).

We wanted to further characterize the effect of zAgRP1 expression on overall adiposity and adipocyte dynamics in living fish. To do this, we first utilized the fluorescent dye 4,4-difluoro-1,3,5,7,8-pentamethyl-4-bora-3a,4a-diaza-s-indacene (BODIPY) to visualize the fat depots in the fish in response to zAgRP1 overexpression. These dyes have been used extensively to study the anatomical distribution and quantity of AT in living zebrafish (Minchin and Rawls, 2017). Upon staining both male and female zebrafish with BODIPY, we found increased overall adiposity in zAgRP1-overexpressing fish compared to that in wild-type controls (Fig. 2A,B). Further analysis into the visceral and subcutaneous fat demonstrated that zAgRP1 specifically increased the area of AT in the visceral abdominal region and not the subcutaneous tail depot (Fig. 2C-F). This demonstrates that our *Tg(-3.5ubb:zAgRP1-2A-EGFP)* fish model represents clinical aspects of obesity, as the disease pathology is more strongly associated with excess visceral fat (Fox et al., 2007; Goodpaster et al., 2003).

### Agrp results in increased susceptibility to high-fat diet (HFD)

Obesity has both a genetic and environmental component, and it is thought that patients with mutations in the melanocortin signaling pathway exhibit poor dietary control by preferring foods with a high-fat content (van der Klaauw et al., 2016). Similarly, studies in mice demonstrate that alterations in this pathway promote increased preference for HFDs (Koegler et al., 1999; Tung et al., 2007). Because Agrp plays a role in regulating adiposity as well as HFD-seeking behaviors, we wanted to determine the effect of zAgRP1 overexpression in zebrafish on adipocyte dynamics in the context of an HFD. To better visualize these dynamics (compared to BODIPY), we utilized a previously developed zebrafish line in which the adipocytes lipid droplets are fluorescently labeled with a Plin2-tdTomato construct (Lumaquin et al., 2021) and are highly sensitive to HFD. These *casper* fish were developed by injecting a Plin2-tdTomato fusion protein at the one-cell stage and outcrossed to create a stable line. Plin2 is a perilipin protein that surrounds the lipid droplets in cells. The fluorescently tagged protein predominantly labels adipocytes in fish, allowing for *in vivo* tracking of adipocyte dynamics (Lumaquin et al., 2021). We injected the *ubb:zAgRP1-2A-EGFP* construct into the *Tg(-3.5ubb:plin2-tdTomato)* line and outcrossed several generations to generate a stable line (Fig. 2G). We then took 21 days post-fertilization (dpf) larvae and fed them either a commercially available HFD or control diet for a week and measured visceral adiposity using tdTomato fluorescence (Fig. 2H). We found that, in response to an HFD, zAgRP1 fish had increased adipocyte expansion compared to that of wild-type controls (Fig. 2I; Fig. S4A).

In order to confirm that this effect is conserved when fish are adults, we fed the F3 zAgRP1 fish an HFD for 3 months and then sent them for histology. We found that male zAgRP1 fish that were fed an HFD had larger adipocytes than those of male zAgRP1 fish on a control diet or wild-type fish on a control diet or HFD (Fig. 2J; Fig. S5A). There was no combinatorial effect on AT area (Fig. S5C). In contrast, this effect was not present in female fish, which were predominantly affected by an HFD (Fig. 2J; Fig. S5B). Further investigation demonstrated that the combination of HFD and zAgRP1 altered liver histology in both male and female fish, with a greater effect observed in females (Fig. S5G). We did not see a synergistic effect of HFD and zAgRP1 on adult fish weight over several weeks (Fig. S5A,B). Overall, these findings demonstrate that zAgRP1-expressing fish are more susceptible to HFD-induced obesity, with males having greater susceptibility than females. Our findings on the obesity-related criteria across our models are summarized in Table S1.

### Agrp increases melanoma onset in male zebrafish

**Fig. 3.**
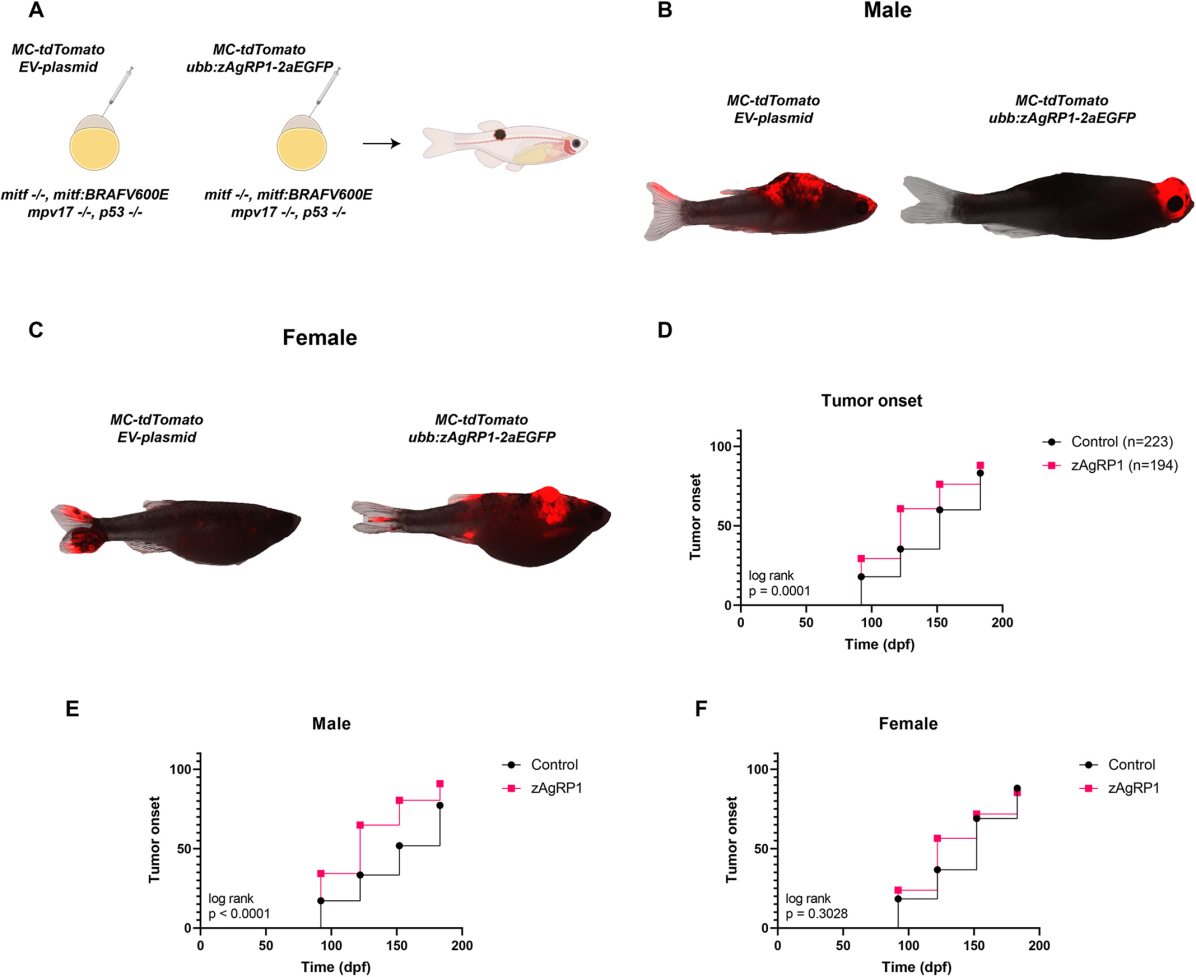
**Agrp overexpression increases tumor onset in an embryo injection model of melanoma.** (A) Schematic of embryo injection experiment to determine tumor onset with and without zAgRP1. Created with Biorender.com. (B,C) Representative images of male (B) and female (C) fish with tumors from empty vector (EV) control or zAgRP1 overexpression. (D) Tumor onset of MiniCoopR-rescued male and female fish combined. (E,F) Tumor onset of MiniCoopR-rescued male (E) and female (F) fish separated from D. Fish were injected at the one-cell stage and monitored for tumors at the indicated time points starting at 3 months post-fertilization (mpf). Data are the averages of three biological replicates. Log-rank (Mantel-Cox) .

Given the obesogenic effects of both Agrp and HFD, we then studied this in the context of melanoma. As described above, the transgenic zebrafish had a latent *BRAF^V600E^* gene in the germline, which can be activated by injection of the MiniCoopR plasmid, as previously described (Iyengar et al., 2012). The MiniCoopR plasmid contains a *mitfa* minigene driven by the *mitf* promoter. It also contains tdTomato driven by the *mitfa* promoter. Injection of the MiniCoopR construct into the *mitfa*^−/−^ *casper* fish rescues *mitfa* in a mosaic fashion. Because these fish also contain the latent BRAF^V600E^, expression of *mitfa* activates the oncogene in tdTomato^+^ cells and rescues melanocytes (Iyengar et al., 2012). We co-injected MiniCoopR-tdTomato with either *ubb:zAgRP1-2A-EGFP* or an empty vector construct (Fig. 3A). Similar to in the experiments above, the fish that were injected with *ubb:zAgRP1-2A-EGFP* were larger than the empty vector controls (Fig. 3B,C). Mosaic expression of GFP was also variable across fish (Fig. S6A,B). We screened the fish for tumors starting at 2 mpf, imaged the fish starting at 3 mpf, and assessed melanoma onset using a previously established rubric developed in the laboratory (Weiss et al., 2022). These criteria are based on the combination of hyperpigmentation, tdTomato fluorescence and growth of lesions into or out of the fish. This showed that the zAgRP1-overexpressing obese zebrafish developed tumors significantly faster than empty vector controls (Fig. 3D). When we segregated these fish by sex and calculated tumor onset, we found increased onset in male fish but not female fish (Fig. 3E,F). These data are the first animal studies to demonstrate the clinically observed sex-specific effect of obesity on melanoma (Sergentanis et al., 2013).

### Agrp increases tumor initiation and area in Rb1 mutant melanoma

Although these data clearly demonstrate that zAgRP1 overexpression leads to increased tumor onset, there are several limitations to this model in which the transgenes are injected at the embryonic stage. In this model, tumorigenesis occurs in the embryo, and tumor onset manifests over time. This is an issue for our particular question as zebrafish do not start differentiating their sex until ∼20 dpf and do not display any reliable sex phenotypes until at least 1 mpf, reaching reproductive maturity at 2.5-3 mpf (Kossack and Draper, 2019). Additionally, zAgRP1 overexpression induces overfeeding, which is not detected in weight until 4 mpf, long after the transgene is activated. It is also hard to detect differences in tumor size in this model, or to study metastasis, because the transgene is expressed everywhere, and we cannot discern multifocal primary tumors versus metastatic lesions. To better address these issues, we instead turned to an electroporation-based method called Transgene Electroporation in Adult Zebrafish (TEAZ) (Callahan et al., 2018), in which plasmids are directly electroporated into adult skin cells. Thus, tumor onset occurs somatically in adulthood (similar to humans), is spatially and temporally controlled, and can be monitored for tumor area and metastasis occurrence.

**Fig. 4.**
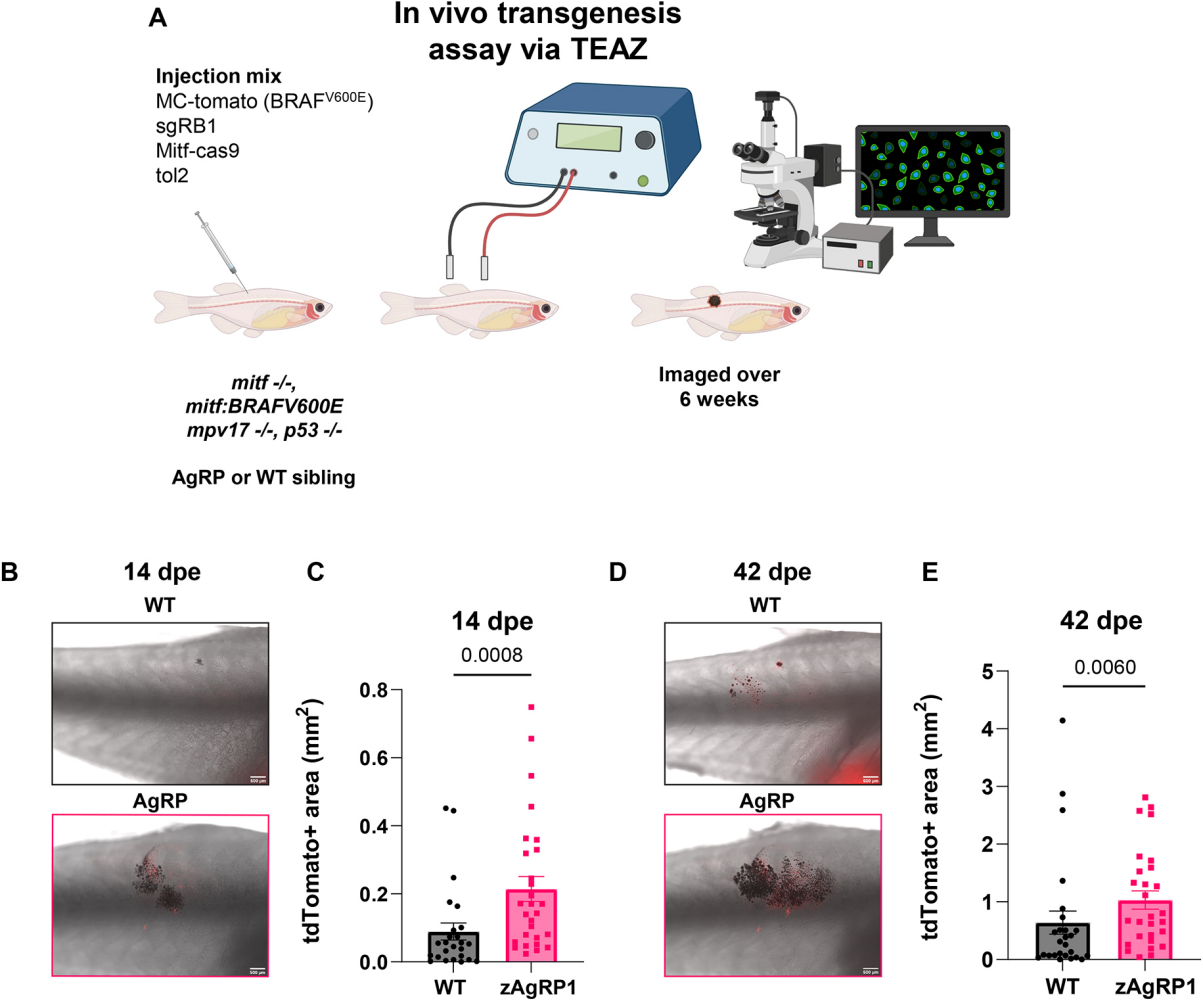
**Obesity increases tumor initiation and area in an electroporation model of Rb1 mutant melanoma.** (A) Schematic of *in vivo* transgenesis assay via Transgene Electroporation in Adult Zebrafish (TEAZ). Adult *casper* (*mitfa:*BRAF^V600E^*, p53^−/−^, mitfa^−/−^, mpv17^−/−^*) zAgRP1 or wild-type F0 fish were injected with MiniCoopR-tdTomato, sgRB1, mitfa:Cas9 and tol2 constructs and then electroporated. Fish were analyzed for tumor initiation and area by fluorescence microscopy over 6 weeks. Created with Biorender.com. (B,C) Tumor area at 14 days post-electroporation (dpe). Representative images (B) and quantification (C) of wild-type and zAgRP1 fish. (D,E) Tumor area at 42 dpe. Representative images (D) and quantification (E) of wild-type and zAgRP1 fish. *n*≥25 per genotype. Data are the averages of three biological replicates. Mann–Whitney test. Scale bars: 500 μm.

We used TEAZ to initiate tumors driven by BRAF^V600E^;*p53*^−/−^;*rb1*^−/−^ in fish with or without zAgRP1 overexpression (Fig. 4A). This was accomplished by electroporating the MiniCoopR plasmid with additional constructs including a *mitfa*-driven Cas9 and a U6-gRNA against Rb1. This approach has previously been shown to induce melanoma development and is responsive to genetic perturbations (Baggiolini et al., 2021; Tagore et al., 2021 preprint). We found that zAgRP1 increased tumor initiation detected at 14 days post-electroporation (dpe) as well as tumor area of early (14 dpe; Fig. 4B,C) and late (42 dpe; Fig. 4D,E) lesions. We found that these lesions were relatively slow growing and rarely developed metastasis. Therefore, we sought to determine the effect of zAgRP1 on a more aggressive model by swapping the Rb1 deletion for a Pten deletion (Fig. S7A). This resulted in tumors that were more aggressive, growing faster and larger than those in the Rb1 deletion model (Fig. S7B,C), a phenomenon that has previously been reported in mice (Dankort et al., 2009). We also observed more variability in the size of the tumors at the end of the study in the Pten mutant tumors, although there was some variability across both groups (Fig. S7B,C). Interestingly, there was no significant effect of zAgRP1 layered on top of a Pten deletion (Fig. S7D-G). This was the case for both early and late lesions.

### Obesity increases tumor initiation in Rb1 mutant melanoma in male zebrafish

**Fig. 5.**
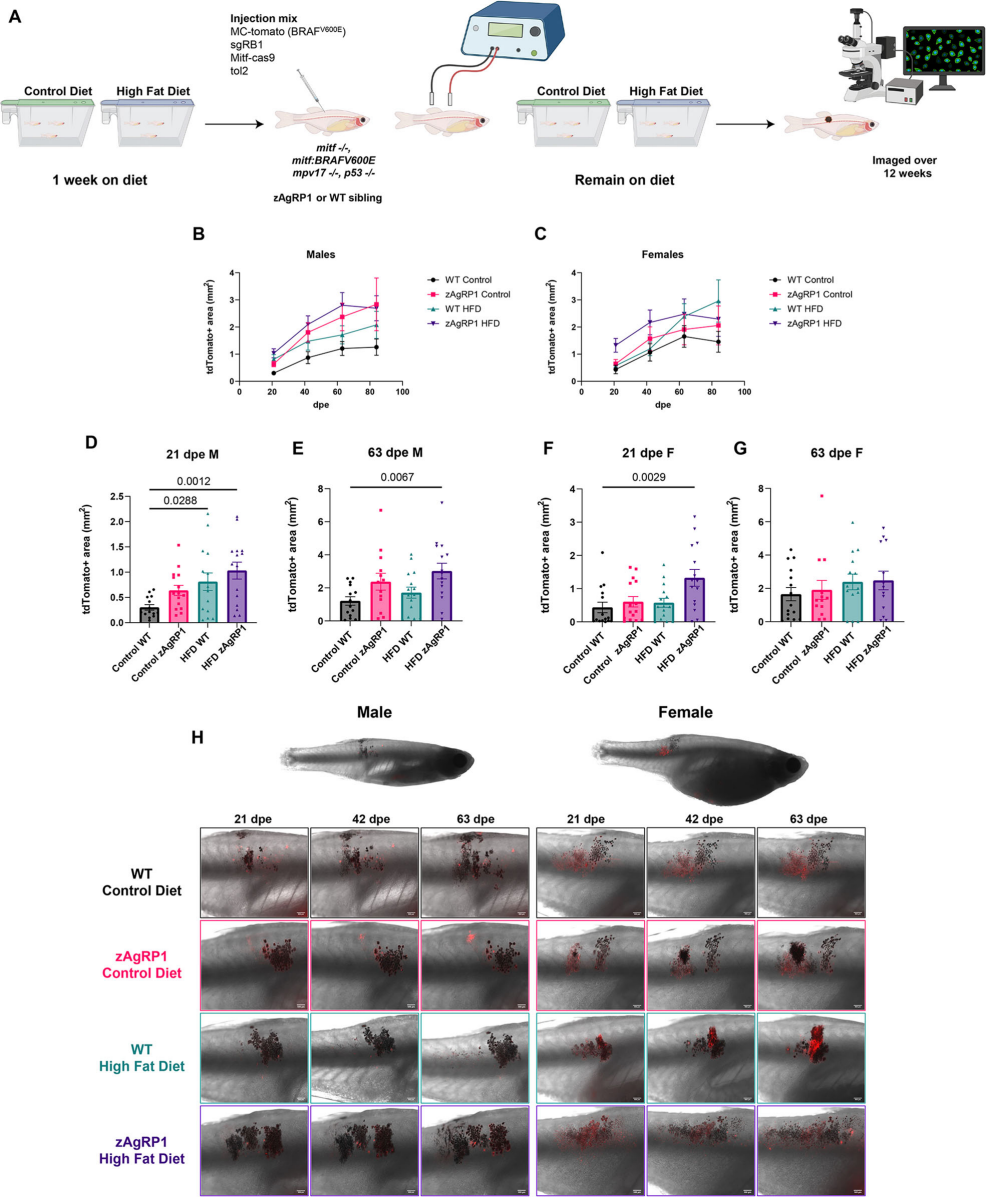
**Obesity increases tumor growth in a sex-dependent manner in Rb1 mutant melanoma.** (A) Schematic of *in vivo* transgenesis assay via TEAZ with the addition of an HFD. Fish were put on a control diet or HFD for 1 week. Adult *casper* (*mitfa:*BRAF^V600E^, *p53^−/−^, mitfa^−/−^, mpv17^−/−^*) zAgRP1 or wild-type F3 fish were injected with MiniCoopR-tdTomato, sgRB1, mitfa:Cas9 and tol2 constructs and then electroporated. Fish were analyzed for tumor initiation and area by fluorescence microscopy over 12 weeks while they remained on their respective diets. Created with Biorender.com. (B,C) Tumor growth as measured by tdTomato^+^ area over time. TdTomato^+^ area of lesions on zAgRP1 or wild-type male (B) and female (C) fish at indicated dpe. Growth curves were analyzed via mixed-effects analysis, with *P*=0.0344 significance when comparing the different conditions in males and *P*=0.2391 in females. (D,E) tdTomato^+^ area for early (21 dpe; D) and late (63 dpe; E) lesions in male zAgRP1 or wild-type fish on either a control diet or HFD. (F,G) tdTomato^+^ area for early (21 dpe; F) and late (63 dpe; G) lesions in female zAgRP1 or wild-type fish on either a control diet or HFD. (H) Representative fluorescence overlaid on brightfield images of lesions in male and female fish at the indicated time dpe. Representative images were chosen based on numerical values closest to the mean for each condition. *n*=14 fish per condition per sex. Data are the averages of three biological replicates. Dunnett's multiple comparisons test. Scale bars: 500 μm.

Because the TEAZ transgenesis assay allows us to induce a tumor in a fully immunocompetent adult animal, we next sought to extend our findings on obesity and melanoma in the context of zAgRP1 overexpression or HFD. We also investigated whether this was dependent on sex. We used the *Tg(-3.5ubb:zAgRP1-2A-EGFP)* and wild-type siblings and fed them an HFD for 1 week based on our data that zAgRP1 can induce changes in 1 mpf larvae after a week on the diet (Fig. 2H,I). We then introduced the BRAF^V600E^ mutation in conjunction with a Rb1 deletion into the fish using TEAZ and monitored fish for tumors via fluorescence microscopy over the course of 12 weeks (Fig. 5A). During this time, the fish remained on their respective diets. This revealed a significant sex-based difference in tumor growth (Fig. 5B,C). In male fish, all obesity conditions (genetic, diet or combined) led to tumors that were larger than those of wild-type control diet fish (Fig. 5B). When observing early (21 dpe; Fig. 5D,H) and late (63 dpe; Fig. 5E,H) individual time points, we observed that the effect was stronger earlier on and seemed to become less apparent at later stages, suggesting that the effect is primarily on tumor initiation rather than on progression. In contrast, we did not observe in females a significant difference in growth curves between the wild-type fish on a control diet or HFD and zAgRP1 fish on either diet (Fig. 5C). Furthermore, there was no difference at early (21 dpe; Fig. 5F,H) or late (63 dpe; Fig. 5G,H) time points. However, the combination of genetic- and diet-induced obesity did have an additive effect on tumor area only at early time points (21 days). This was not seen in later lesions, further underscoring lack of increased tumor area in response to obesity specifically in females.

### Obesity-related effects on melanoma are not seen with Pten

Finally, we determined whether this was maintained with a different genetic driver (Fig. S8A). We therefore studied the effect of BRAF^V600E^;*p53*^−/−^;*ptena/b*^−/−^. Unlike the case for BRAF^V600E^;*p53*^−/−^;*rb1*^−/−^, we did not see a significant difference in the tumor growth in the context of a Pten deletion in both male and female fish (Fig. S8B,C). This was true in both early and late lesions (Fig. S8D-H). As expected, the tumors with a Pten deletion were larger and grew faster than those with an Rb1 deletion. Overall, these data demonstrate that the interaction between systemic alterations (i.e. diet, sex) and the tumor is dependent on the genotype of the tumor itself (Pten versus Rb1 deletion). This highlights that it is critical to develop models in which it is possible to study multiple facets in both the host and the tumor concurrently. The flexibility of the zebrafish system will allow future studies to dissect these interactions in a high-throughput manner.

## DISCUSSION

In this study, we aimed to develop the zebrafish as a new model for studying the interaction between obesity and melanoma, and whether this depends on somatic and germline factors. This revealed a sex-specific effect on melanoma initiation and tumor onset in tumors initiated by BRAF^V600E^;*p53*^−/−^;*rb1*^−/−^ but not in those initiated by BRAF^V600E^;*p53*^−/−^;*ptena/b*^−/−^. Our results are consistent with clinical literature on human patients that demonstrates an increased hazard ratio for melanoma in males (Dobbins et al., 2013; Sergentanis et al., 2013). In females, we did not observe any significant difference in response to either Agrp or an HFD, which is similar to the clinical reports that there is no association between obesity and melanoma risk in females (Dobbins et al., 2013; Sergentanis et al., 2013). This is in contrast to the findings that the effects of obesity on melanoma are present in both sexes (Gallus et al., 2006; Gogas et al., 2008) or the reports of no association between the diseases (Præstegaard et al., 2015). Our results suggest a role for sex in the complex association between obesity and melanoma.

Although studies such as these are more traditionally done using mouse models of cancer, the rapidity by which we can generate these perturbations, and precisely quantify tumor initiation and progression, make the zebrafish an ideal system for future studies. The major advance offered in this system is the ease by which we can make somatic mutations (i.e. loss of Rb1 or Pten) using the TEAZ system, which is far simpler and faster than comparable mouse models. Although the TEAZ method is still variable from animal to animal, the larger sample size we can generate allows us to discern even subtle signals. Moreover, these can be readily combined with germline alterations in genes such as *AGRP*, a key member of the most frequently altered pathway seen in patients with obesity, or with dietary changes such as HFDs.

One of the important observations in our research is that obesity has a sex-specific effect on melanoma initiation, with obesity having a greater effect in males than in females. However, one key limitation of our study is that the Agrp/HFD model does not produce all of the obesity phenotypes seen in humans, and not all measures of obesity tracked the same way in male and female fish. As summarized in Table S1, we used multiple different criteria to assess ‘obesity’ in our fish, but these phenotypes are likely to be found only in subsets of human patients. For example, male fish have increased weight but not BMI, whereas female fish have increased BMI. Males have an increase in both total AT area and adipocyte size, whereas females only have an increase in adipocyte size. We saw no change in subcutaneous adiposity, but only in visceral adiposity in both sexes, which may relate to the liver steatosis we see in our model, as has been reported in humans (Gentile et al., 2015). Agrp alters blood metabolites in each group; however, in male zebrafish, the increase was in blood glucose, whereas in female zebrafish it was in TGs, both of which have been seen to be elevated in obese humans (Wakabayashi and Daimon, 2012). However, sex differences in these blood metabolites in relation to obesity in patients are more complex (Mittendorfer et al., 2016). For example, blood glucose is elevated in both sexes in response to obesity, but is observed at a lower BMI in males than in females. Additionally, females' blood glucose is more correlative with BMI than males’ blood glucose, and diabetic women are more obese than diabetic men (Kautzky-Willer et al., 2016).

These limitations make it difficult to directly ascribe a mechanism by which Agrp increases melanoma growth specifically in male fish, i.e. we do not know which of the obesity-related phenotypes we observed in the fish (weight versus adipocyte size versus BMI, etc.) are most tightly linked to the melanoma phenotypes we have observed. It is probable that Agrp and HFD affect melanoma in both cell-intrinsic and cell-extrinsic ways. For example, prior work has shown that cancer-associated adipocytes can contribute to tumor progression in a variety of diseases such as melanoma (Zhang et al., 2018), breast cancer (Dirat et al., 2011) and ovarian cancer (Nieman et al., 2011). Whether the obesogenic adipocytes we observe here from Agrp contribute to melanoma pathogenesis in this cell non-autonomous way needs further investigation. Another area that has come under investigation is the lipid droplets themselves, which are formed when cancer cells take up fatty acids. For example, the key lipid droplet protein DGAT1 promotes melanoma growth, and our data using the Plin2 lipid droplet reporter suggest that this is likely increased in the Agrp animals (Wilcock et al., 2022). Furthermore, how melanoma pathogenesis is affected by the intersection of sex and obesity is poorly understood at a mechanistic level.

Despite numerous human and animal studies demonstrating that males are at higher risk of developing melanoma to begin with (Pandey et al., 2012; Renehan et al., 2008; Sergentanis et al., 2013), it has also long been observed that females have improved survival once they have the disease (Hieken et al., 2020; White, 1959). The mechanisms regulating this discrepancy remain poorly understood. One potential reason includes a stronger immune responsiveness in females (Bouman et al., 2005; Gubbels Bupp et al., 2018). Hormonal influences are also thought to play an important, yet still not understood, role. Estrogen binds to multiple receptors with somewhat opposing effects. Estrogen receptor α (ERα) is thought to be pro-proliferative, whereas estrogen receptor β (ERβ) may act in an anti-proliferative manner, and men were found to have comparatively lower levels of ERβ (de Giorgi et al., 2013). Estrogens can also bind to the more recently described G protein-coupled estrogen receptor (GPER), which can inhibit melanoma growth and is thought to mediate some of the protective effect in females (Natale et al., 2018). Testosterone likely also plays a role, because melanomas are known to express androgen receptors (ARs), with dihydrotestosterone thought to be especially mitogenic (Richardson et al., 1999). Adding to this complexity is the so-called ‘obesity paradox’, in which obesity can be associated with improved survival specifically in males. For example, a large retrospective meta-analysis showed that obese males have improved response to targeted or immunotherapy treatments (McQuade et al., 2018). Although our studies do not address the mechanisms involved in these seemingly contradictory effects on initiation versus survival, there is evidence that polymorphisms of p53 may play a role in such sex-specific effects (Oliveira et al., 2017). The fact that we see this sex difference in the context of p53/Rb1, but not p53/Pten, also highlights a potential interaction between germline alleles and somatic mutations in the PTEN/PI3K pathway. This is particularly compelling given the known interactions between ERβ and the PI3K pathway (Dey et al., 2013; Lei et al., 2020). We also do not know the effect of the *mpv17* mutation in our *casper* zebrafish model, which could be relevant for obesity given that it is an inner mitochondrial membrane protein (D'Agati et al., 2017; Krauss et al., 2013; Spinazzola et al., 2006). These would be fruitful areas for future exploration using the zebrafish system we have developed.

Beyond tumor initiation, the role of sex and obesity in melanoma progression, metastasis and drug resistance is another area that our models could be used for. There is evidence that there is an association between obesity and melanoma aggressiveness, as obesity is associated with thickness of primary cutaneous melanoma (Skowron et al., 2015). Furthermore, several mouse xenograft models of melanoma demonstrate increased tumor volume in diet or genetic models of obesity (Pandey et al., 2012; Ringel et al., 2020; Brandon et al., 2009). Our studies suggest an increase in tumor initiation based on a decrease in response in late lesions, and future studies should be aimed at looking at endpoints such as metastasis. Another potential future application of our model is in understanding response to therapies. Recent work has demonstrated that females have improved survival in response to BRAF/MEK (MAP2K) inhibitors compared to males, in part due to AR expression being elevated in males post-treatment. Combination therapy of BRAF/MEK inhibitors with AR blockade leads to improved responses in both sexes (Vellano et al., 2022). Because obesity leads to a sex-specific improved response to therapy in melanoma (McQuade et al., 2018), and melanoma itself has a sex-dependent response to therapy, future studies should focus on manipulating melanoma cells directly in lean and obese fish. This is an area that our Agrp zebrafish model in conjunction with TEAZ can directly address.

## MATERIALS AND METHODS

### Zebrafish husbandry

All zebrafish (*Danio rerio*) were bred and housed in the Aquatics facility in Zuckerman Research Center at Memorial Sloan Kettering Cancer Center. All fish were housed in standard conditions with a water temperature of 28.5°C, controlled salinity, a pH of 7.4 and a light/dark cycle of 14 h/10 h. Unless otherwise indicated, fish were initially fed rotifers followed by a standard commercial pellet diet (GEMMA) three times per day. Fish were housed at a density no higher than ten fish per liter. Anesthesia of adult zebrafish was carried out using tricaine (4 g/l, Syndel, Ferndale, WA, USA) that was diluted to 0.16 mg/ml. All of the protocols listed in this manuscript were reviewed and approved by the Memorial Sloan Kettering Cancer Center Institutional Animal Care and Use Committee, protocol #12-05-008.

### Zebrafish transgenic lines

The zebrafish lines used in these studies were the *casper* (*mitfa:*BRAF^V600E^*, p53^−/−^, mitfa^−/−^, mpv17^−/−^*) fish (Baggiolini et al., 2021; Tagore et al., 2021 preprint) and the *Tg(-3.5ubb:plin2-tdTomato)* line (Lumaquin et al., 2021). These lines were generated in the White laboratory previously. For this study, we also generated the *Tg(-3.5ubb:zAgRP1-2A-EGFP)* and *Tg(-3.5ubb:plin2-tdTomato,-3.5ubb:zAgRP1-2A-EGFP) casper* fish lines as described below.

### Generation of Agrp construct

To generate the *ubb:zAgRP1-2A-EGFP* construct, Gateway Cloning, using LR Gateway Enzyme mix (Thermo Fisher Scientific, Waltham, MA, USA; #11791019), was completed using a p5E-*ubb*, pME-zAgRP1, p3E-2A-EGFP into the pDestTol2pA2-blastocidin destination vector. The control plasmid, an empty pDestTol2pA2-blastocidin vector, was generated from colonies that arose in the destination vector control reaction. All selected colonies were cultured overnight and prepared using a HiSpeed Plasmid Maxi Kit (Qiagen, Hilden, Germany; #12663) and sequenced at Genewiz (South Plainfield, NJ, USA) for confirmation. To create the pME-zAgRP1 construct for gateway cloning, the cDNA sequence was obtained from Song et al. (2003) and ordered as a gBlock Gene Fragment (IDT DNA, Coralville, IA, USA), PCR amplified using AccuPrime Taq DNA Polymerase High Fidelity (Invitrogen, Waltham MA, USA; #12346086), and gel extracted using a NucleoSpin Gel and PCR Clean-up kit (Takara Bio, San Jose, CA, USA; #740609). The fragment was subsequently cloned into the pME vector using a pENTR/D-TOPO Cloning kit (Invitrogen; #K240020). Colonies were prepared using a Qiaprep Spin Miniprep kit (Qiagen; #27106) and sequenced at Genewiz for confirmation.

### Generation of zAgRP1 fish

In order to generate the *Tg(-3.5ubb:zAgRP1-2A-EGFP) casper* (*mitfa:*BRAF^V600E^*, p53^−/−^, mitfa^−/−^, mpv17^−/−^*) mosaic fish, *ubb:zAgRP1-2A-EGFP* and Tol2 mRNA were injected into the yolk of *casper* (*mitfa:*BRAF^V600E^*, p53^−/−^, mitfa^−/−^, mpv17^−/−^*) fish at the one-cell stage (Baggiolini et al., 2021; Tagore et al., 2021 preprint). Fish were sorted for GFP^+^ fluorescence by the 2aEGFP and used for mosaic experiments or to generate a stable line of F2 and F3 fish. For mosaic experiments, uninjected quad fish from the same clutch were used as control. To make the stable line, a single F0 male fish was outcrossed to a single female *casper* (*mitfa:*BRAF^V600E^*, p53^−/−^, mitfa^−/−^, mpv17^−/−^*) fish. Several pairs were set up, and fish from independent clutches were utilized. Fish were outcrossed for two generations to quad zebrafish, and GFP^−^ siblings were used as the wild-type control.

### Real-time quantitative PCR (RT-qPCR)

Zebrafish were euthanized in tricaine, and bulk tissue was dissected from the dorsum of the zebrafish. Tissue was snap frozen in liquid nitrogen. RNA was harvested from the bulk tissue as directed by the manufacturer (Zymo Research, Irvine, CA, USA; #R1055). To generate cDNA, 25-50 ng RNA was transcribed to cDNA using a Superscript III First-Strand Synthesis System (Invitrogen; #18080051). For qPCR, cDNA was diluted 1:10 and used as the initial template in Power SYBR Green PCR Master Mix (Thermo Fisher Scientific; #4368708). The primers used were as follows: *hatn10* fw, 5′-TGAAGACAGCAGAAGTCAATG-3′; *hatn10* rv, 5′-CAGTAAACATGTCAGGCTAAATAA-3′; agrp1 fw, 5′-ATGAGGATCTGGGCAAAGCTGT-3′; *agrp1* rv, 5′-GAAGGCCTTAAAGAAGCGGCAGTA-3′ (Opazo et al., 2018). Gene expression levels after DNA amplification in a Bio-Rad CFX384 Real-Time detection system were measured and normalized to a reference gene, *hatn10*, using ΔCt values.

### Determination of blood metabolites

Blood was collected from zebrafish as previously described (Babaei et al., 2013). Briefly, zebrafish were anesthetized in tricaine. Immediately after the heart stopped beating, the tail was severed, and fish were placed in a collection tube pierced on the bottom within a falcon tube and centrifuged at 500 ***g***. Both the collection tube and the Eppendorf were coated with 500 IU heparin. Blood was then spun down, and plasma was collected. Glucose (Abcam, Cambridge, UK; ab65333) and TGs (Abcam; ab65336) were quantified using commercially available kits. Values were normalized to protein content in the plasma measured via a Bradford assay.

### Generation of zAgRP1 Plin2tdTomato fish

In order to generate the *Tg(-3.5ubb:plin2-tdTomato, −3.5ubb:zAgRP1-2A-EGFP) casper* mosaic fish, 25 ng/μl *ubb:zAgRP1-2A-EGFP* and 20 ng/μl Tol2 mRNA were injected into the yolk of *Tg(-3.5ubb:plin2-tdTomato) casper* fish at the one-cell stage (Lumaquin et al., 2021). Fish were sorted for both overall GFP^+^ fluorescence and GFP^+^ hearts, then used to generate a stable line of F2 and F3 fish. To make the stable line, F0 fish were outcrossed two generations to *Tg(-3.5ubb:plin2-tdTomato) casper* zebrafish, and GFP^−^ body, but GFP^+^ heart, siblings were used as the wild-type control.

### Determination of zebrafish weight and length

Adult fish were anesthetized with tricaine and then dried using a paper towel. Length was determined to the closest 0.01 mm using electronic calipers (Cole-Parmer, Vernon Hills, IL, USA; #EW-09925-43), and weight was determined to the nearest 0.01 g using a portable balance (OHAUS, Parsippany, NJ, USA; #SPX222). Fish were then placed into a tank with fresh system water to recover.

### Histology

Zebrafish were sacrificed using ice-cold water. The head and tail were removed, and fish were placed in 4% paraformaldehyde (PFA) in PBS for 72 h at 4°C on a rocker. Fish were then transferred to 70% EtOH for 24 h at 4°C on a rocker. Fish were sent to Histowiz (Brooklyn, NY, USA), where they were paraffin embedded, sectioned coronally at 5 μm and underwent H&E staining.

### IHC

IHC for Plin2 was conducted at Histowiz. Paraffin-embedded sections were probed for Plin2 using a commercial antibody at a dilution of 1:200 (Proteintech, Rosemont, IL, USA; #15294-1-AP).

### HFD feeding

For HFD experiments, fish were fed a commercially developed pelleted fish food (Sparos, Olhão, Portugal) (Lumaquin et al., 2021). Both the HFD and matched control diets were developed by Sparos. The crude composition as per fed basis for the control diet was 57.3% crude protein, 13.1% crude fat, 0.5% fiber, 8.8% ash and 20.3 MJ/kg gross energy. The crude composition as per fed basis for the HFD was 57.3% crude protein, 24.8% crude fat, 0.5% fiber, 8.8% ash and 23.3 MJ/kg gross energy. The diets were analyzed for final composition. The Sparos control diet contains 30% fishmeal, 33% squid meal, 5% fish gelatin, 5.5% wheat gluten, 12% cellulose, 2.5% soybean oil, 2.5% rapeseed oil, 2% vitamins and minerals, 0.1% vitamin E, 0.4% antioxidant, 2% monocalcium phosphate and 2.2% calcium silicate. The Sparos HFD contains 30% fishmeal, 33% squid meal, 5% fish gelatin, 5.5% wheat gluten, 12% palm oil, 2.5% soybean oil, 2.5% rapeseed oil, 2% vitamins and minerals, 0.1% vitamin E, 0.4% antioxidant, 2% monocalcium phosphate and 2.2% calcium silicate. For the larvae experiments, 21 dpf larvae were moved to 0.8 l tanks in equal density (15-20 larvae). Fish were fed 0.1 g of food split 2× per day. Fish were kept on the diet for 1 week and then imaged for visceral adiposity via tdTomato expression. The images were analyzed, and the visceral adiposity was measured using MATLAB (Lumaquin et al., 2021). HFD-induced expansion was calculated as the tdTomato^+^ area of the HFD-fed group divided by the average of the control-fed group for the indicated genotype. For adult fish experiments, fish were fed 5% of body weight per fish split 2× per day. Fish were kept on the diet for indicated time points (1-3 months).

### Embryo injection transgenesis and analysis

In order to generate melanomas using embryo injection-based transgenics, we used the MiniCoopR-based transgenic model as previously described (Ceol et al., 2011; Kaufman et al., 2016). Fish were injected at the one-cell stage with 15 ng/μl MiniCoopR-tdTomato, 15 ng/μl of either *ubb:zAgRP1-2A-EGFP* or empty vector control, and 20 ng/μl Tol2 mRNA. Fish were monitored at 48 h post-fertilization for GFP and tdTomato fluorescence. Fish were put in the nursery at 5 dpf, and then monitored for tumors starting at 2 mpf and imaged at 3 mpf. Fish were checked monthly for tumors up until 6 mpf. Tumors were determined based on a rubric of criteria developed in the laboratory previously (Weiss et al., 2022). The bases of the criteria are hyperpigmentation, tdTomato fluorescence and growth into or out of the fish. Disease-free survival curves were generated using Prism 8 (GraphPad, San Diego, CA, USA).

### TEAZ-based transgenesis and analysis

Melanomas were generated using TEAZ as described previously (Callahan et al., 2018). All tumors generated had a *BRAF^V600E^* mutation and loss of *p53* with the additional loss of either *rb1* or *ptena* and *ptenb*. To generate these tumors, fish were anesthetized with 0.16 mg/ml tricaine and injected with 1 μl *rb1* tumor plasmid mix (250 ng/μl *mitfa:*Cas9, 250 ng/μl MiniCoopR-tdTomato, 106 ng/μl sgRB1, 67 ng/μl Tol2) or *ptena/b* tumor plasmid mix (250 ng/μl *mitfa:*Cas9, 250 ng/μl MiniCoopR-tdTomato, 27.3 ng/μl gPTENa, 27.3 ng/μl gPTENb, 61 ng/μl Tol2) into the skin below the dorsal fin. Fish were then electroporated and returned to fresh system water to recover. Electroporation was carried out using a CM 830 Electro Square Porator (BTX Harvard Apparatus, Holliston, MA, USA) and 3 mm platinum Tweezertrodes (BTX Harvard Apparatus; #45-0487). The electroporator settings were LV mode with a voltage of 40 V, five pulses, 60 ms pulse length and 1 s pulse interval. Electroporated zebrafish were imaged serially for up to 12 weeks post-electroporation using brightfield and fluorescence imaging. Area of tdTomato fluorescence was quantified at indicated time points using FIJI.

### Imaging and analysis

Zebrafish were imaged using an upright Zeiss AxioZoom V16 Fluorescence Stereo Zoom Microscope equipped with a motorized stage, brightfield and fluorescent filter sets (mCherry or Cy5, GFP and tdTomato). Adult fish were imaged with a 0.5× adjustable objective lens, and larvae were imaged with a 1.0× adjustable objective lens. To acquire images, zebrafish were lightly anesthetized with 0.16 mg/ml tricaine. Images were acquired with Zeiss Zen Pro v2 and exported as CZI files for visualization and analysis. Imaging analysis was completed manually using FIJI or automated with MATLAB software (MathWorks, Natick, MA, USA).

### Statistical analysis

All experiments were completed at least three times as independent biological experiments. Statistical analysis was completed using Prism versions 8 and 9 (GraphPad). Exact sample sizes and statistical tests for each experiment are detailed in the figure legends. Data are presented as mean±s.e.m. Statistical significance was set at *P*≤0.05.

## Supplementary Material

10.1242/dmm.049671_sup1 Supplementary information
