# Supplementary material for: Modeling the effects of genetic- and diet-induced obesity on melanoma progression in zebrafish
Source: Dis Model Mech. 2023 Jan 20;16(1):dmm049671. doi: 10.1242/dmm.049671 (PMC9884122; doi:10.1242/dmm.049671)
Supplement: Supplementary information [file dmm-16-049671-s1.pdf]

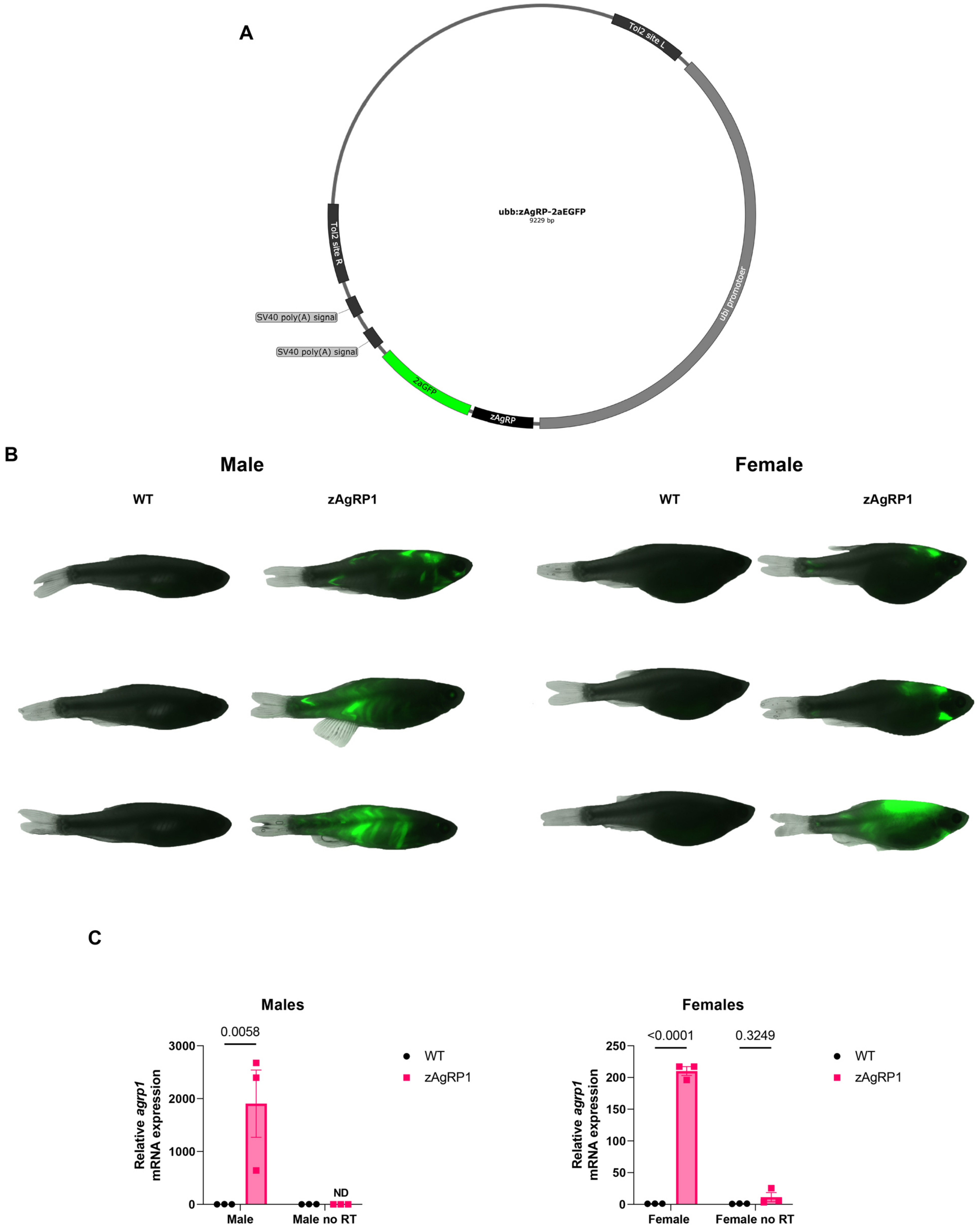

Fig. S1 related to Figure 1. zAgRP overexpression in casper zebrafish. (A) AgRP overexpression construct driven by the ubb promoter. Created with Snapgene. (B) Representative images of GFP expression from F0 mosaic fish. (C) agrp1 mRNA expression in adult F3 zebrafish. RNA was harvested from bulk tissue from male (left panel) and female (right panel) adult fish. RNA was transcribed to cDNA and analyzed by qPCR. No RT = no Reverse transcriptase. ND = not detected. Two-way ANOVA.

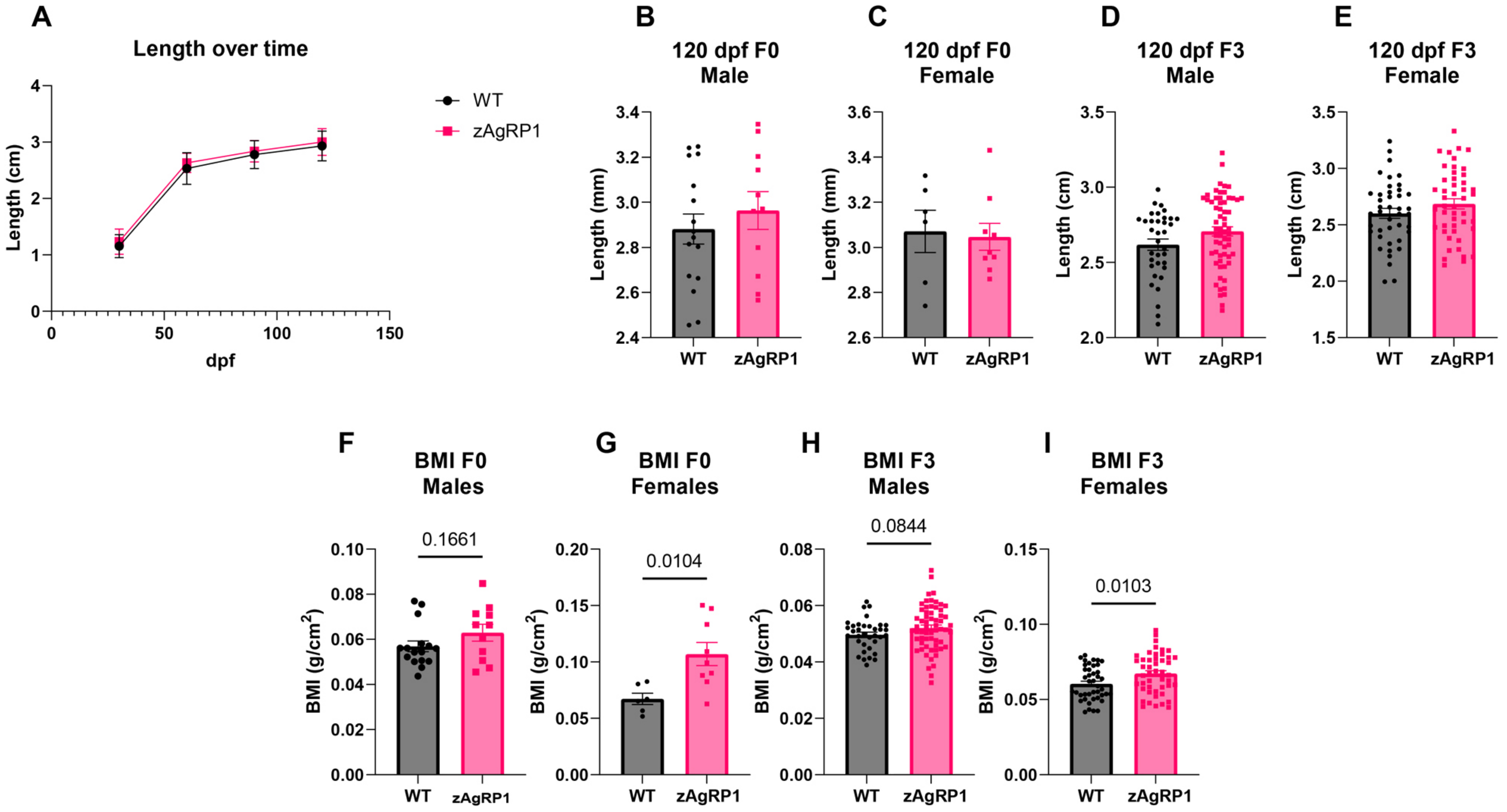

Fig. S2. related to Figure 1. zAgRP1 overexpression does not change the length of zebrafish. (A) Combined length of both male and female F0 fish over 6 months. (B-C) Length of male (B) and female (C) fish at 120 dpf of F0 mosaic fish. Fish were separated into equal numbers at one month and length measured at indicated time points. n=5-10 fish per genotype per biological replicate. Data is the average of 3 biological replicates. (D-E) Length of male (D) and female (E) fish at 120 dpf of F3 stable line. The data is the weight of all the fish from 2 clutches from separate single male and female pairs of fish across six separate tanks. n=35-60 fish per group. (F-G) BMI of male (F) and female (G) fish at 120 dpf of F0 mosaic fish. (H-I) BMI of male (H) and female (I) fish at 120 dpf of F3 stable line. BMI was calculated as weight in grams divided by length in centimeters squared. zAgRP1 fish and wild type siblings were housed in the same tanks and identified via GFP fluorescence. Welch's t test.

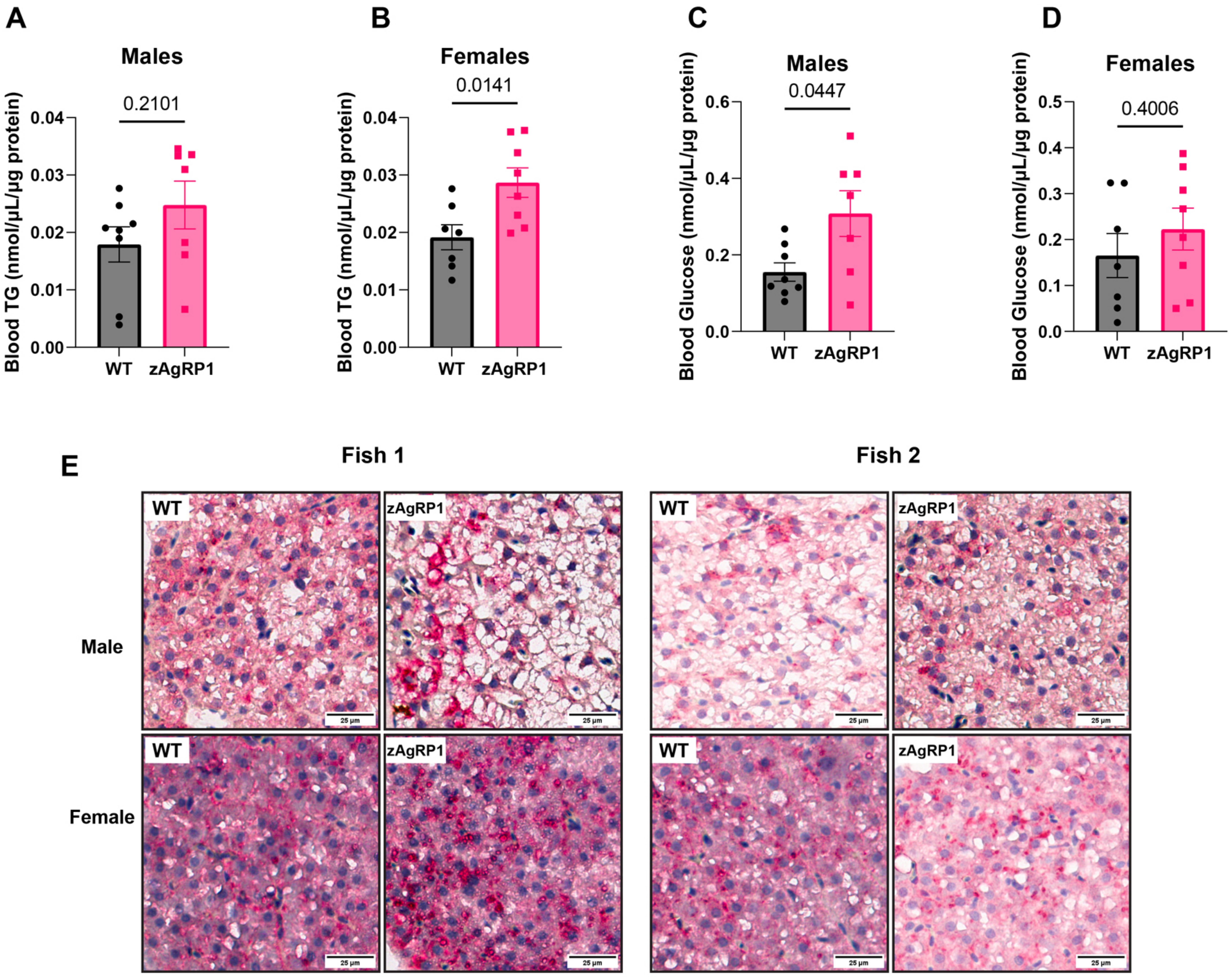

Fig. S3 related to Figure 1. zAgRP1 overexpression alters systemic metabolism. (A-B) Plasma triglyceride (TG) levels from male (A) and female (B) F3 adult fish. (C-D) Plasma glucose from male (C) and female (D) F3 adult fish. Blood was collected by low grade centrifugation and plasma extracted. Blood metabolites were measured and quantified using fluorescent assays. Blood samples were collected from 3 fish per genotype per condition. Samples were run in triplicate and averaged for each assay. The assay was run twice on two independent days. (E) IHC of plin2 protein in the liver of adult male (left) and female (right) adult F3 fish. 2 fish were sent per sex per genotype for IHC staining. Welchꞌs t test.

Disease Models & Mechanisms • Supplementary information

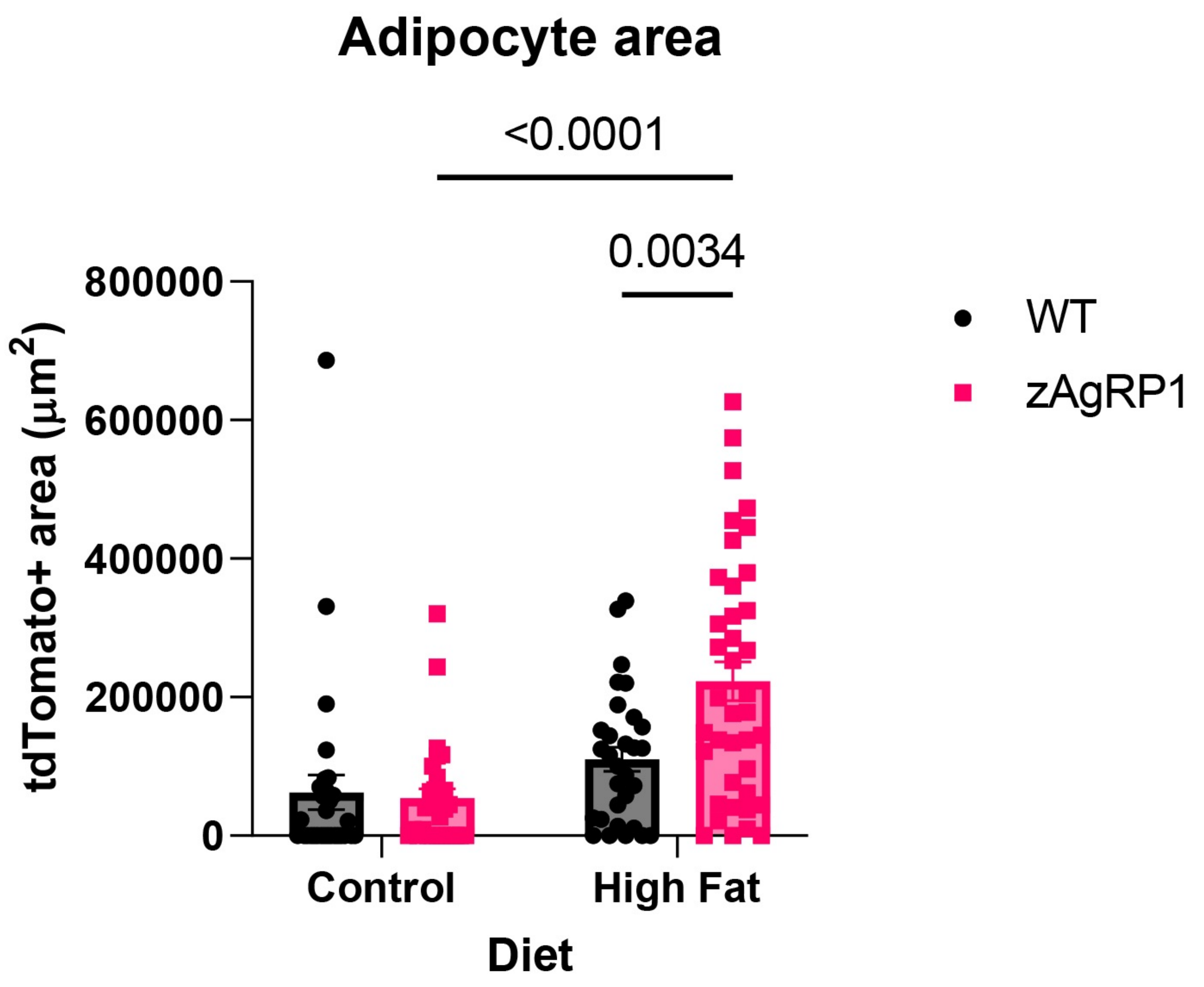

Fig. S4 related to figure 2. Adipocyte area increases in response to HFD to a greater extent in zAgRP1 fish. tdTomato+ area from 21 dpf zAgRP1 or wild type plin2tdTomato fish put on either a control or high fat diet for one week. n ≥ 30 fish per genotype across 3 biological replicates. Two-way ANOVA.

Disease Models & Mechanisms • Supplementary information

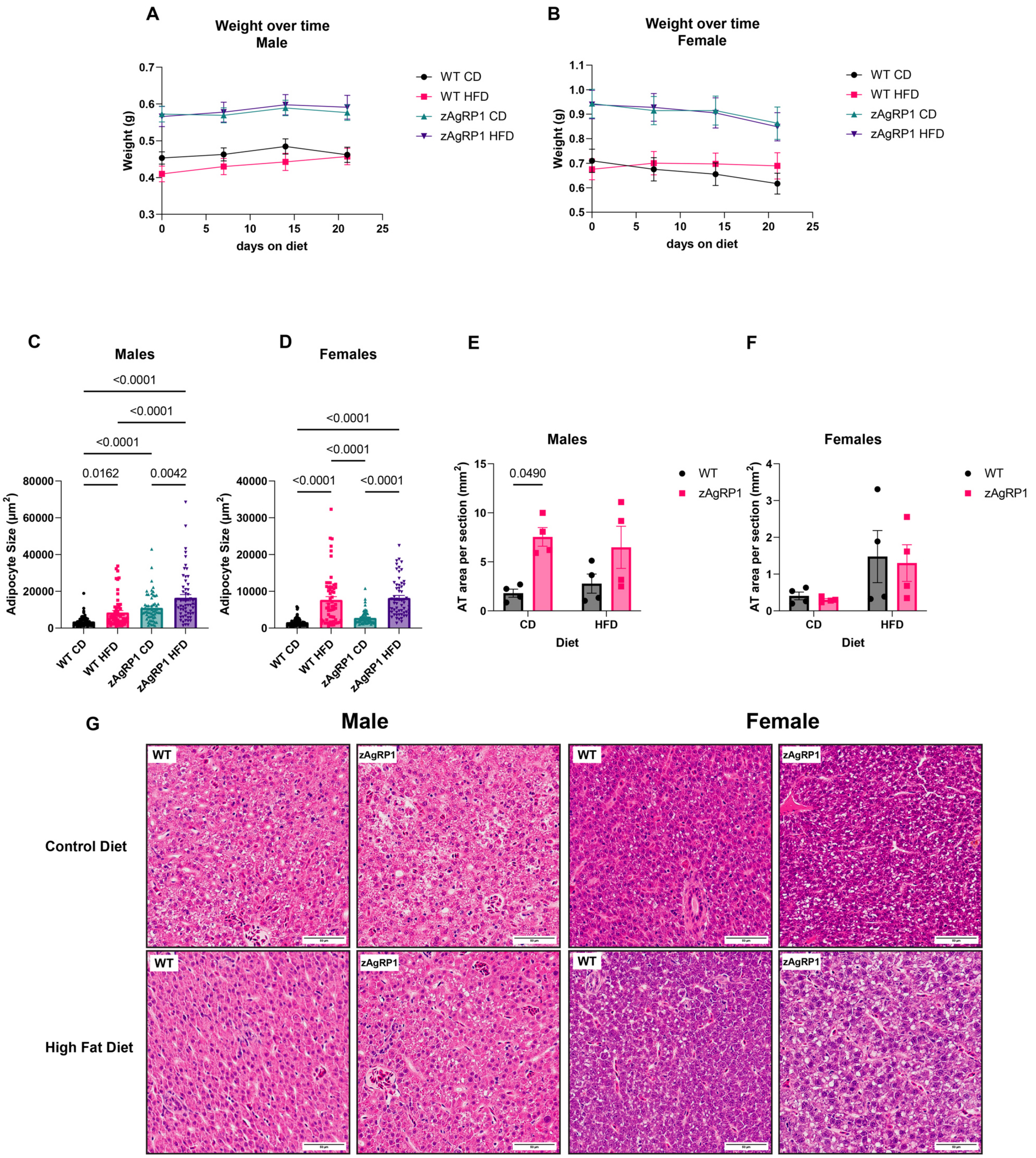

Fig. S5 related to Figure 2. Combination of AgRP and HFD have a combinatorial effect on histology of adult zebrafish (A-B) Weight of male and female adult casper (mitfa:BRAFV600E, p53-/-, mitfa-/-, mpv17-/-) fish with zAgRP1 or wild type controls on a high fat or control diet for 3 weeks. Data is the average of 3 independent biological experiments. n=10 fish per group per sex for each experiment. (C-D) Adipocyte size from male (C) and female (D) adult casper (mitfa:BRAFV600E, p53-/-, mitfa-/-, mpv17-/-) fish with zAgRP1 or wild type controls on a high fat or control diet for 3 months. (E-F) AT area per section from male (E) and female (F) fish. (G) Histology of the liver of male and female adult casper (mitfa:BRAFV600E, p53-/-, mitfa-/-, mpv17-/-) fish with zAgRP1 or wild type controls on a high fat or control diet for 3 months. 2 fish per condition per sex were sent for sectioning.

Disease Models & Mechanisms • Supplementary information

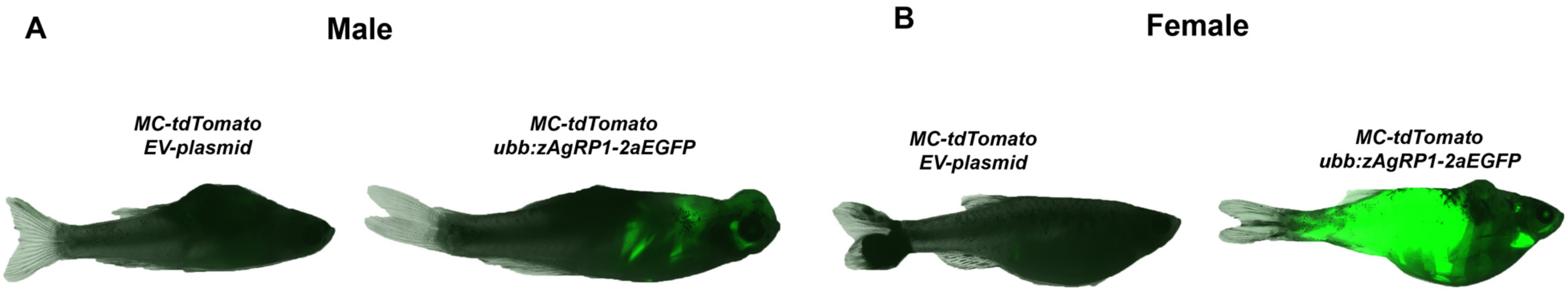

Fig. S6 related to Figure 3. GFP expression in F0 mosaic fish. (A-B) GFP expression overlaid on brightfield images from representative images of F0 mosaic male (A) and female (B) fish in embryo injection model of melanoma.

Disease Models & Mechanisms • Supplementary information

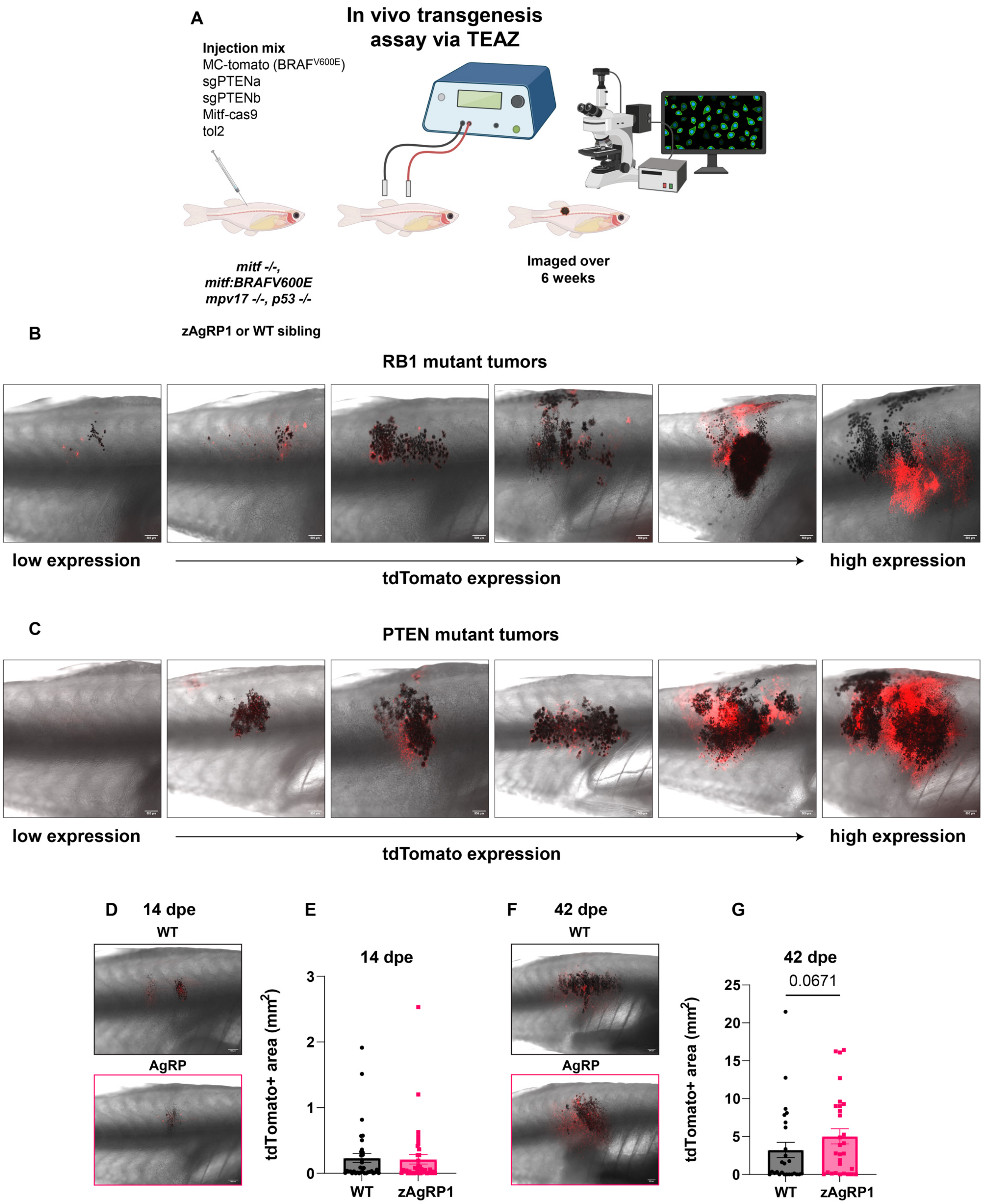

Disease Models & Mechanisms • Supplementary information

Fig. S7 related to Figure 4. Obesity does not increase tumor initiation and area in an electroporation model of PTEN mutant melanoma (A) Schematic of in vivo transgenesis assay via TEAZ. Adult casper (mitfa:BRAFV600E, p53-/-, mitfa-/-, mpv17-/-) zAgRP1 or wild type F0 fish were injected with MiniCoopR-tdTomato, sgPTENa, sgPTENb, mitfa:Cas9, and tol2 constructs and then electroporated. Fish were analyzed for tumor initiation and area by fluorescence microscopy over 6 weeks. Created with Biorender. (B-C) Examples of end-point tumors from RB1 (B) and PTEN (C) mutant melanomas. Images are arranged from lowest to highest tdTomato expression to demonstrate variability from each of the mutant drivers. (D-E) Tumor area at 14 dpe. Representative images (D) and quantification (E) of wild type and zAgRP1 fish. (F-G) Tumor area at 42 dpe. Representative images (F) and quantification (G) of wild type and zAgRP1 fish. n ≥ 26 per genotype. Data is the average of 3 biological replicates. Mann-Whitney test.

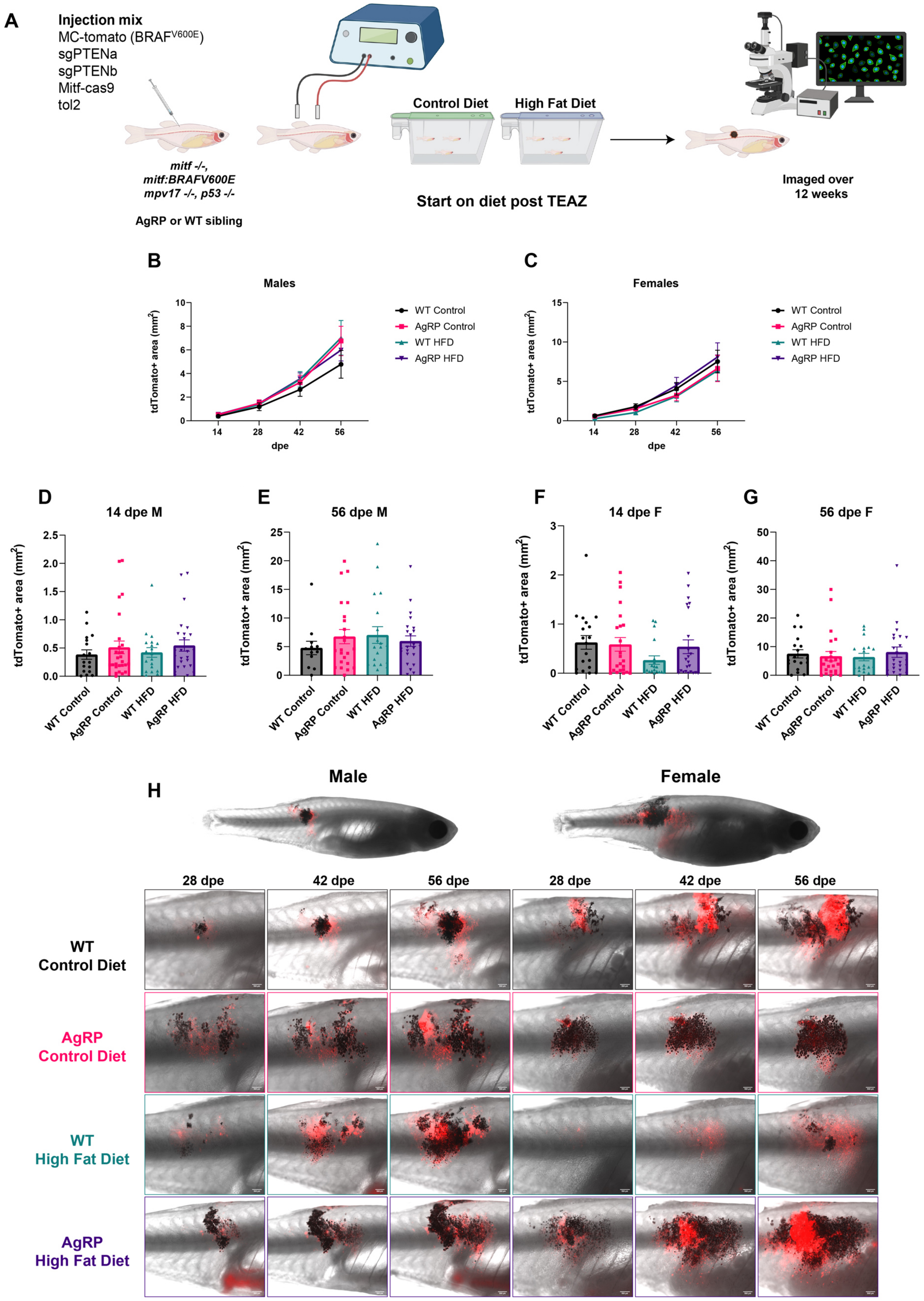
A
Injection mix
MC-tomato (BRAF V600E)
sgPTENa
sgPTENb
Mitf-cas9
tol2
mitf -/-, mitf:BRAFV600E mpv17 -/-, p53 -/-
AgRP or WT sibling
Control Diet
High Fat Diet
Start on diet post TEAZ
Imaged over 12 weeks
B
Males
C
Females
tdTomato+ area (mm2)
dpe
WT Control
AgRP Control
WT HFD
AgRP HFD
D
14 dpe M
E
56 dpe M
F
14 dpe F
G
56 dpe F
H
Male
Female
28 dpe
42 dpe
56 dpe
WT Control Diet
AgRP Control Diet
WT High Fat Diet
AgRP High Fat Diet

Fig. S8 related to Figure 5. Obesity has no effect on tumor growth in sex dependent manner in PTEN mutant melanoma (A) Schematic of in vivo transgenesis assay via TEAZ with the addition of a high fat diet. Adult casper (mitfa:BRAFV600E, p53-/-, mitfa-/-, mpv17-/-) zAgRP1 or wild type F3 fish were injected with MiniCoopR-tomato, sgPTENa, sgPTENb, mitfa:Cas9, and tol2 constructs and then electroporated. Fish were put on a control or high fat diet for the remainder of the experiment. Fish were analyzed for tumor initiation and area by fluorescence microscopy over 12 weeks while they remained on their respective diets. Created with Biorender. (B-C) Tumor growth as measured by tdTomato+ area over time. TdTomato+ area of lesions on zAgRP1 or wild type male (B) and female (C) fish at indicated dpe. Growth curves were analyzed via Two-way ANOVA analysis, with $p=0.5303$ when comparing the different conditions in males and $p=0.6326$ in females. (D-E) tdTomato+ area for early (14 dpe, D) and late (56 dpe, E) lesions in male zAgRP1 or wild type fish on either a control or high fat diet. (F-G) tdTomato+ area for early (14 dpe, F) and late (56 dpe, G) lesions in female zAgRP1 or wild type fish on either a control or high fat diet. (H) Representative fluorescence overlaid on brightfield images of lesions in male and female fish at indicated time dpe. Representative images were chosen based on numerical values closest to the mean for each condition. $n \geq 17$ fish per condition per sex and is the average of 3 biological replicates. Individual time points were analyzed by Dunnet's multiple comparisons test.

Table S1. Obesogenic response to AgRP in male and female zebrafish. List of criteria of obesity and response to AgRP overexpression in both male and female zebrafish. Yes indicates an increase, No indicates unchanged. Glc: Glucose, TG: Triglycerides.

Measure of Obesity	Male	Female
**Weight**	Yes (F3, not significant F0)	Yes
**Length**	No	No
**BMI**	No	Yes
**Visceral Adipose**	Yes	No
**Area Adipocyte Size**	Yes	Yes
**Blood Metabolites**	Yes (Glc), No (TG)	Yes (TG), No (Glc)
**Liver Steatosis**	Some	Yes
